# A Nonsynonymous *FCER1B* SNP is Associated with Risk of Developing Allergic Rhinitis and with IgE Levels

**DOI:** 10.1038/srep19724

**Published:** 2016-01-21

**Authors:** Gemma Amo, Jesús García-Menaya, Paloma Campo, Concepción Cordobés, M Carmen Plaza Serón, Pedro Ayuso, Gara Esguevillas, Miguel Blanca, Jose A.G. Agúndez, Elena García-Martín

**Affiliations:** 1 Department of Pharmacology, Universidad de Extremadura, Cáceres, Spain; 2 Allergy Service, University Hospital Infanta Cristina, Badajoz, Spain; 3 Allergy Service, Hospital Carlos Haya, Málaga, Spain; 4 Research Laboratory, IBIMA-Málaga University General Hospital, Málaga, Spain

## Abstract

Allergic rhinitis is associated with elevated serum IgE levels. IgE response is mediated by the high-affinity IgE receptor (FcεRI), which is polymorphic. Studies analyzing the association between allergic rhinitis and FcεRI variants have been conducted with controversial results. The objective of this study is to analyze, in 1,041 individuals, the putative clinical association of allergic rhinitis with common polymorphisms in FcεRI subunits genes. These SNPs included *FECR1A* rs2494262, rs2427837 and rs2251746; *FECR1B* rs1441586, rs569108 and rs512555; *FCER1G* rs11587213, rs2070901 and rs11421. Statistically significant differences were observed for the *FCER1B* rs569108 and rs512555 polymorphisms frequencies when comparing patients with allergic rhinitis without asthma and controls. The OR (95% CI) value for the 237Gly allele (rs569108) is equal to 0.26 (0.08–0.86, P = 0.017) and for the G allele (rs512555) it is equal to 0.27 (0.08–0.88, P = 0.020). These two SNPs are linked (D’ = 1.0, LOD = 56.05). Also observed was a statistically significant trend towards lower IgE values among allergic rhinitis patients with variant alleles for both SNPs. In conclusion, in patients with allergic rhinitis without asthma, the *FCER1B* rs569108 and rs512555 polymorphisms are associated with increased risk of developing allergic rhinitis and with lower IgE levels.

Allergic rhinitis is associated to family history of allergy^1^,^2^, thus suggesting that genetic risk factors may underlie the risk of developing, or the clinical presentation of, allergic rhinitis^3^,^4^,^5^. Allergic rhinitis is also associated to elevated serum IgE levels and increased mediator release from activated inflammatory cells. IgE cross-link allergens and bind the high-affinity IgE receptor (FcεRI) on the surface of mast cells or basophils, releasing vasoactive mediators, such as histamine. FcεRI on these cells is a heterotetramer of three subunits: FcεRIα, FcεRIβ y FcεRIγ (αβγ_2_) encoded by three genes designated as *FCER1A*, *FCER1B* and *FCER1G*, respectively^6^,^7^. FcεRIα is the ligand-binding subunit, FcεRIβ is a signal-augmenting subunit and FcεRIγ is a signal-transducing subunit^8^. In other cells, such as antigen-presenting cells including monocytes, dendritic cells or macrophages, FcεRI is expressed in a trimeric form lacking the β-subunit^9^,^10^. It also has been reported that platelets, epidermal Langerhans cells or eosinophils can express the receptor on their cellular surface in a trimeric form^11^,^12^.

Although the search for genetic susceptibility factors related to allergic rhinitis is a promising field, gene variations related to FcεRI as potential risk factors for allergic rhinitis have not been comprehensively analyzed, and the results available are in some cases contradictory^5^,^13^,^14^,^15^,^16^,^17^. The genes coding for all FcεRI subunits are polymorphic. Some of these polymorphisms affect IgE levels and/or have been associated with atopic diseases^18^,^19^,^20^,^21^,^22^,^23^. The *FCER1A* gene is located on chromosome 1q23 (1:159259504-159278014)^24^ and it encodes a protein with two extracellular IgE-like domains^7^. The *FCER1B* gene is located on chromosome 11q12-13. The gene spans approximately 10 kilobases (11:59855734-59863444)^24^, and it encodes a 244-amino acid protein with a non-canonic intracellular ITAM domain with an extra tyrosine. FcεRI can be expressed without the β-chain, but when FcεRIβ is present, receptor expression is higher^7^. The *FCER1G* gene is located on chromosome 1q23 (1:161185024-161190489)^24^. The gene encodes an 86-amino acid protein. FCεR1γ plays an essential role in the induction of mast cell degranulation and survival^25^.

Aiming to elucidate the putative association of common gene variants affecting the FcεRI subunits in the clinical presentation and/or in risk of developing allergic rhinitis, we analyzed the association of the variants of *FCER1A*, *FCER1B, FCER1G* genes with several clinical phenotype parameters, including IgE levels, in a large group of well-phenotyped allergic rhinitis patients and control individuals; we also analyzed putative gene-gene interactions which, as yet, have not been explored.

## Results

The SNP frequencies observed in the study group are consistent with those previously described in the 1000 genomes public database for individuals with European descent^24^, and are at Hardy-Weinberg equilibrium. The genotype and allele frequencies for the nine SNPs analyzed in overall allergic rhinitis patients and control individuals are summarized in Table 1. No major frequency differences were observed when comparing patients and controls.

Patients were stratified in two subgroups according to their clinical presentation; allergic rhinitis alone and allergic rhinitis + asthma. Table 2 shows the *FCER1* genotypes. Among patients with allergic rhinitis alone the frequencies for individuals carrying the minor alleles for the SNPs rs569108 and rs512555 were remarkably reduced among allergic rhinitis patients as compared to the control individuals. In fact, only three allergic rhinitis patients (representing 2% of this clinical subgroup) carried such minor alleles in heterozygosity, whereas 37 controls (7.2%) carried such variant alleles. It is to be noted that no carriers of these variant alleles in homozygosity were observed among all patients and controls (Table 1). Statistical significance was also observed in the test for trend for the SNPs rs569108 and rs512555 (χ^2^ = 5.86, P = 0.015; χ^2^ = 5.60, P = 0.018, respectively). The SNP rs569108 corresponds to a missense variant E237G, which is predicted as deleterious by Sorting Intolerant From Tolerant (SIFT)^26^, and predicted as possibly damaging by the Polymorphism Phenotyping v2 (PolyPhen-2) prediction^27^. The SNP rs512555 is a downstream gene variant whose functional effect is unknown; the high linkage between these two SNPs in the present study (D’ = 1.0, LOD = 56.05) suggests that the genetic association observed in Table 2 might actually be related to the missense SNP rs569108. The rest of the SNPs analyzed did not reveal any differences either in genotypes or in allele frequencies, when patients with allergic rhinitis alone and control individuals were compared (Table 2). Among patients with allergic rhinitis + asthma none of the SNPs analyzed revealed significant differences when compared to control individuals (Table 2).

Because it has been suggested that *FCER1* genotypes may modify IgE response^21^, we determined IgE values and analyzed their distribution according *FCER1* genotypes. These findings are summarized in Table 3. A statistically significant trend can be observed towards lower IgE values among patients with variant alleles for the SNPs rs569108 and rs512555, confined to patients with allergic rhinitis alone. Among patients with allergic rhinitis + asthma who carried the above-mentioned variant alleles, the IgE values were lower as compared to non-carriers, but the difference was not statistically significant. The rest of the variant alleles were not related to the IgE levels.

Since a statistical significant association of the *FCER1B* variant alleles with IgE levels in patients with allergic rhinitis alone was detected, linear regression under the standard additive model, including in a single model all genotypes, gender, age, familial antecedents of allergic diseases, smoking and prick tests, was studied with regard to the IgE values. Only age, with lower IgE concentrations in older age, (p = 0.019) and the mutated genotypes corresponding to the *FCER1B* SNPs rs569108 and rs512555 genotypes (p = 0.005) remained significant. The rest of the putative genetic associations were discarded in the multiple analysis. No associations of genotypes or IgE levels were identified when individuals were stratified with regard to allergic rhinitis type (intermittent, persistent mild and persistent moderate), eosinophilia, FEV or asthma severity step.

## Discussion

Allergic rhinitis, like other complex diseases, results from interplay of genetic and environmental risk factors^3^,^4^,^5^. The heritability factor has been calculated to be quite high, between 74–82%^1^ and 90%^2^, and several genetic variants are known to be associated with allergic rhinitis risk in some populations^3^,^28^,^29^. However, associations have been described with genes which have a weak, or unknown, relation with allergic rhinitis pathogenesis, and it has been speculated that the putative associations could be due to hitherto unknown epigenetic mechanisms^3^. In contrast, the present study focuses on genes that are functionally related to the pathogenesis and the clinical presentation of this disease.

Our results show association of two SNPs in the *FCER1B* gene with the risk of developing allergic rhinitis: the nonsynonymous Glu237Gly (rs569108) and the 3′UTR SNP (rs512555). The Glu237 allele is more common among allergic rhinitis alone patients than in healthy subjects. When allergic rhinitis + asthma patients were compared to healthy subjects, we observed no statistically significant differences in the allele frequencies or genotypes. The *FCER1B* Glu237Gly variant has been studied in a Japanese population (233 patients with nasal allergy and 100 control subjects) showing an association between Gly 237 and nasal allergy and atopy^30^. Recently, a meta-analysis including these two polymorphisms^16^ has been published. Such study includes 4496 asthmatics and 4571 controls for the E237G variant and 2005 cases and 1868 controls for rs1441586 from several populations: Caucasians, Africans and Asians. The results obtained suggest that the 237Gly allele might influence the risk for developing atopic asthma, although no significant association was observed in the total population. Regarding the rs1441586 SNP, a significant association with asthma risk was observed among Asians only^16^. Our findings do not support such an association with asthma, but they do support association with rhinitis. To date, no meta-analyses with allergic rhinitis patients have been performed. The close relationship between asthma and allergic rhinitis is well known. A majority of adult patients with asthma also suffer from allergic rhinitis^17^ and vice-versa (this study). However, the presence or absence of allergic rhinitis + asthma can be an important confounding factor in the studies carried out so far, which did not discriminate these clinical presentations. In this sense careful phenotyping using strictly defined guidelines should be used to elucidate whether the putative associations with *FECR1* polymorphisms are related to asthma or to concomitant allergic rhinitis. In this sense, we cannot rule out that environmental factors may modify the observed associations. Further PheWas studies would be necessary to elucidate this point.

The functional mechanism underlying the association found in our study is a matter of speculation, and there are certain aspects which should be taken into consideration. The regulation of the expression of FcεRI is crucial to the receptor function. It is known that both the expression of FcεRIβ subunit and the serum levels of IgE are important regulating factors. FcεRIβ regulates the receptor response to IgE through its capacity to amplifying receptor signaling. IgE- FcεRI binding causes overexpression of the receptor^18^,^20^ with an increase in the total FcεRIα content on the surface. This process can be reverted by the removal of IgE, which results in a reduction in receptor expression and FcεRIα content^19^. The relation between *FCER1B* variants and IgE levels is controversial. A wide variation in the IgE levels, consistent with previous studies, was observed in the present study^31^,^32^. We observed that the *FCER1B* variants are associated with lower serum IgE levels, in a group of patients phenotyped to discriminate between patients with allergic rhinitis without asthma and allergic rhinitis + asthma. The association is lost when all allergic rhinitis patients are merged as a single clinical group, as has been done in most previous studies. Another source of variability with previous findings is that the variant alleles analyzed in this study show different frequencies in diverse ethnicities^24^; these differences could explain, at least in part, the different results described to date.

Several studies have reported an association between several *FCER1A* variants and total serum IgE levels^21^ as well as increased risk for atopic eczema and asthma. We failed to replicate the previously described association of IgE levels with the *FCER1A* polymorphism rs2427837, although it is important to note that this association was described in a specific population corresponding to a general population of individuals living in Augsburg^21^. Another study in a Han Chinese population failed to find an association between *FCER1A* variants and IgE level or allergic allergic rhinitis susceptibility^22^.

Summing up, we have identified an association of *FCER1B* variants with the risk of developing allergic rhinitis alone, and with IgE levels in these patients. Further association studies using well-phenotyped patients are required to identify genetic associations that, so far, may have been overlooked in studies considering heterogeneous groups of patients.

## Methods

### Study Population

We studied a cohort of 1,041 individuals, composed of 515 unrelated patients with allergic rhinitis and 526 unrelated healthy controls. Written consent for participation was obtained for all participants. All the patients and 97% of the control subjects who were invited to participate in the study agreed to do so.

Of the patients, 265 were recruited from the Allergy Department, Infanta Cristina Hospital (Badajoz, Spain) and 250 were recruited from the UGC Allergy, Regional Hospital (Málaga, Spain). Clinical characteristics of the participants are summarized in Table 4. Most of these patients had participated in a previous study and their clinical characterization has been described elsewhere^33^. Diagnosis for allergic rhinitis was based on the diagnostic criteria defined by the Joint Task Force on Practice Parameters in Allergy, Asthma and Immunology^34^. All patients were diagnosed by an allergy specialist and had a positive skin prick test^35^ for one or more aeroallergens. Of the 515 patients with allergic rhinitis, 366 (71.1%) was diagnosed with allergic asthma, according to American Thoracic Society criteria^36^. These patients were classified as defined by the step classification of the Global Initiative for Asthma guidelines^37^. A total of 526 unrelated healthy controls, from the same geographic area, and ethnically matched with patients, were included in the study. Medical history was obtained and examination was performed for each participant to exclude pre-existing disorders. Individuals with familial (up to second-degree relatives) or personal antecedents of allergic, atopic or autoimmune diseases were excluded. Control subjects were selected from staff and from medical students belonging to the Hospitals and the Universities participating in the study.

No statistically significant differences between cases and controls were observed for gender. Age was not different when comparing the overall group of patients and controls (T-Test P = 0.190). However patients with allergic rhinitis alone had a mean age slightly lower than controls (P = 0.035). The percentage of smokers was lower among patients with rhinitis plus asthma (Chi-Square test P = 0.002) as compared to control individuals, whereas the percentage of smokers was higher among patients with allergic rhinitis alone (P = 0.002) as compared to control individuals. The protocol for this study was in accordance with the Declaration of Helsinki and its subsequent revisions, and it was approved by the respective Ethics Committees of the participating Hospitals. All individuals were Spanish Caucasians.

### Genotype analysis

Genomic DNA was obtained from leukocytes and purified according to standard procedures. The selection of the SNPs analyzed was based on allele frequencies in the study population (higher than 0.01), as well as functional or clinical relevance, in accordance with the public 1000 genomes database^24^ and published evidence^38^,^39^. SNP analyses were performed by means of TaqMan assays (Life Technologies, Alcobendas, Spain). Details of these assays are provided in supplemental Table S1. Detection was carried out by qPCR in a 7500 Real Time qPCR system (Life Technologies, Alcobendas, Spain). The amplification conditions were those recommended for the manufacturer. All SNP analyses were carried out in triplicate.

### Statistical analyses

Statistical analyses were carried out by using the Statistical Package for the Social Sciences (SPSS) version 19.0 for Windows (SPSS Inc., Chicago, Ill. USA). Genotype, allelic and Hardy-Weinberg equilibrium analyses were performed by using the software PLINK^40^.

The comparison of the frequencies for alleles and genotypes between patients and control individuals was performed with the χ^2^ statistics or Fisher exact test, as appropriate. The putative association between the SNPs and the risk of developing rhinitis was estimated by determining the odds ratio (OR) and the 95% confidence interval (CI). Multiple comparison adjustments were done by using the False Discover Rate (FDR) correction^41^. The statistical significance of the differences in analytical or clinical parameters were calculated by using the non-parametric Kruskall-Wallis test for independent samples. The gene-dose effect was analyzed by using the test for trend (Cochran-Armitage test). Potential confounders considered in this study were the type of allergic rhinitis, age, gender, familial antecedents of the disease, smoking habits, the presence of asthma and asthma severity step. Multiple analyses were performed by using logistic regression. Differences in the parameters determined were considered to be statistically significant when adjusted p values were lower than 0.05.

In order to calculate the statistical power of the study, we evaluated the sample size with a genetic model based on the frequency for carriers of the risk SNP with an OR value = 2 (α = 0.05). According to the sample size in this study, the statistical power (two-sided associations) for the risk related to the presence of the variant alleles was higher than 99.8% for all SNPs except for the SNPs rs569108 (power = 93.2%) and rs512555 (power = 92.6%).

## Additional Information

**How to cite this article**: Amo, G. *et al.* A Nonsynonymous *FCER1B* SNP is Associated with Risk of Developing Allergic Rhinitis and with IgE Levels. *Sci. Rep.* **6**, 19724; doi: 10.1038/srep19724 (2016).

## Supplementary Material

Supplementary Information

## Figures and Tables

**Table 1:** *FCER1* genotypes in overall allergic rhinitis patients and control individuals.

	Group	Non-mutated (frequency)	Heterozygous (frequency)	Homozygous (frequency)	Minor allele frequency	Comparison Allele frequency OR (95% C.I.); P; Pc
FCER1A						
rs2494262	Patients	0.266	0.476	0.258	0.496	0.93 (0.78–1.12); 0.411; 0.955
	Controls	0.237	0.498	0.265	0.514	
rs2427837	Patients	0.563	0.371	0.066	0.251	0.95 (0.78–1.16); 0.814; 0.955
	Controls	0.539	0.399	0.062	0.262	
rs2251746	Patients	0.553	0.379	0.068	0.257	1.00 (0.82–1.22); 0.872; 0.955
	Controls	0.545	0.395	0.060	0.258	
FCER1B						
rs1441586	Patients	0.279	0.501	0.220	0.471	1.03 (0.87–1.23); 0.500; 0.955
	Controls	0.274	0.526	0.200	0.463	
rs569108	Patients	0.926	0.074	0.000	0.037	0.99 (0.62–1.57); 0.955; 0.955
	Controls	0.925	0.075	0.000	0.037	
rs512555	Patients	0.928	0.072	0.000	0.036	0.99 (0.62–1.57); 0.936; 0.955
	Controls	0.927	0.073	0.000	0.036	
FCER1G						
rs11587213	Patients	0.718	0.262	0.019	0.150	0.96 (0.75–1.22); 0.898; 0.955
	Controls	0.715	0.257	0.027	0.156	
rs2070901	Patients	0.526	0.384	0.089	0.282	0.94 (0.78–1.14); 0.732; 0.955
	Controls	0.512	0.388	0.100	0.294	
rs11421	Patients	0.683	0.286	0.031	0.174	0.94 (0.75–1.17); 0.773; 0.955
	Controls	0.679	0.274	0.047	0.184	

P values correspond to stratified Chi-Square Tests. Pc correspond values adjusted for multiple comparisons according the False Discovery Rate (FDR) procedure. OR: odds ratio. CI: 95% confidence interval.

**Table 2:** *FCER1* genotypes in patients with allergic rhinitis classified according the presence of asthma.

Allergic rhinitis alone	Non-mutated (frequency)	Heterozygous (frequency)	Homozygous (frequency)	Minor allele frequency	Comparison with controls Allele frequency OR (95% C.I.); P; Pc
FCER1A					
rs2494262	0.275	0.503	0.221	0.473	0.85 (0.66–1.10); 0.529; 0.842
rs2427837	0.564	0.403	0.034	0.235	0.87 (0.64–1.17); 0.585; 0.842
rs2251746	0.550	0.403	0.047	0.248	0.95 (0.71–1.28); 0.655; 0.842
FCER1B					
rs1441586	0.315	0.450	0.235	0.460	0.99 (0.76–1.28); 0.209; 0.627
rs569108	0.980	0.020	0.000	0.010	0.26 (0.08–0.86); 0.009; 0.041
rs512555	0.980	0.020	0.000	0.010	0.27 (0.08–0.88); 0.009; 0.041
FCER1G					
rs11587213	0.725	0.262	0.013	0.144	0.91 (0.63–1.31); 0.891; 0.891
rs2070901	0.523	0.409	0.067	0.272	0.90 (0.67–1.20); 0.614; 0.842
rs11421	0.709	0.257	0.034	0.162	0.86 (0.61–1.22); 0.845; 0.891
**Allergic rhinitis +Asthma**	**Non-mutated (frequency)**	**Heterozygous (frequency)**	**Homozygous (frequency)**	**Minor allele frequency**	**Comparison with controls Allele frequency OR (95% C.I.); P; Pc**
FCER1A					
rs2494262	0.266	0.467	0.266	0.500	0.95 (0.78–1.15); 0.416; 0.769
rs2427837	0.569	0.350	0.081	0.256	0.97 (0.78–1.22); 0.511; 0.769
rs2251746	0.560	0.359	0.081	0.260	1.02 (0.81–1.27); 0.598; 0.769
FCER1B					
rs1441586	0.274	0.512	0.214	0.470	1.03 (0.84–1.25); 0.579; 0.769
rs569108	0.901	0.099	0.000	0.050	1.34 (0.83–2.17); 0.461; 0.769
rs512555	0.904	0.096	0.000	0.048	1.33 (0.82–2.16); 0.553; 0.769
FCER1G					
rs11587213	0.716	0.266	0.018	0.151	0.96 (0.74–1.26); 0.881; 0.881
rs2070901	0.527	0.374	0.099	0.286	0.96 (0.77–1.19); 0.762; 0.857
rs11421	0.671	0.302	0.027	0.178	0.96 (0.75–1.24); 0.541; 0.769

**Table 3:** IgE values according to *FCER1* genotypes.

	Genotype	Allergic rhinitis alone	K-W Test across genotypes P; Pc	Allergic rinitis + Asthma	K-W Test across genotypes P; Pc	Overall patients	K-W Test across genotypes P; Pc
FCER1A							
rs2494262	C/C	184.94 ± 232.47	P = 0.139; Pc = 0.250	346.51 ± 482.83	P = 0.560; Pc = 0.937	294.33 ± 424.13	P = 0.935; Pc = 0.972
	C/A	187.68 ± 188.84		267.86 ± 333.20		239.74 ± 293.54	
	A/A	266.87 ± 292.81		306.76 ± 634.82		297.62 ± 555.56	
rs2427837	G/G	194.33 ± 226.68	P = 0.343; Pc = 0.514	300.01 ± 412.43	P = 0.937; Pc = 0.937	265.68 ± 362.08	P = 0.841; Pc = 0.972
	G/A	226.68 ± 241.53		290.18 ± 558.77		267.13 ± 468.99	
	A/A	140.60 ± 118.43		328.59 ± 421.92		298.76 ± 384.97	
rs2251746	T/T	190.06 ± 226.53	P = 0.034; c = 0.102	298.66 ± 413.61	P = 0.915; Pc = 0.937	263.74 ± 368.07	P = 0.372; Pc = 0.837
	T/C	238.70 ± 238.76		297.09 ± 554.46		276.18 ± 466.41	
	C/C	105.86 ± 113.70		302.71 ± 425.47		255.75 ± 376.03	
FCER1B							
rs1441586	T/T	176.95 ± 174.85	P = 0.098; Pc = 0.221	267.91 ± 326.28	P = 0.344; Pc = 0.830	233.06 ± 280.70	P = 0.972; Pc = 0.972
	T/C	236.85 ± 248.41		286.22 ± 399.42		272.75 ± 361.14	
	C/C	181.37 ± 252.96		374.63 ± 738.18		303.91 ± 616.37	
rs569108	A/A	210.43 ± 229.10	P = 0.006; Pc = 0.027	309.51 ± 487.16	P = 0.242; Pc = 0.830	275.40 ± 419.36	P = 0.114; Pc = 0.549
	A/G	11.00 ± 4.58		175.38 ± 165.47		160.91 ± 164.89	
	G/G	–		–		–	
rs512555	C/C	210.43 ± 229.10	P = 0.006; Pc = 0.027	308.11 ± 485.44	P = 0.369; Pc = 0.830	274.67 ± 418.34	P = 0.183; Pc = 0.549
	C/T	11.00 ± 4.58		182.70 ± 166.24		166.91 ± 166.18	
	T/T	–		–		–	
FCER1G							
rs11587213	A/A	200.60 ± 231.02	P = 0.954; Pc = 0.954	310.60 ± 510.95	P = 0.737; Pc = 0.937	273.55 ± 440.24	P = 0.921; Pc = 0.972
	A/G	224.68 ± 229.73		257.38 ± 307.77		249.42 ± 285.10	
	G/G	152.00 ± 80.61		380.17 ± 666.52		323.13 ± 573.94	
rs2070901	G/G	211.39 ± 219.17	P = 0.843; Pc = 0.954	299.32 ± 421.34	P = 0.834; Pc = 0.937	271.91 ± 370.55	P = 0.969; Pc = 0.972
	G/T	187.57 ± 181.20		312.20 ± 576.70		267.26 ± 476.60	
	T/T	279.13 ± 489.95		247.19 ± 244.88		254.28 ± 307.33	
rs11421	T/T	201.13 ± 232.38	P = 0.918; Pc = 0.954	269.59 ± 464.41	P = 0.089; Pc = 0.801	274.04 ± 401.77	P = 0.163; Pc = 0.549
	T/C	225.03 ± 232.90		338.47 ± 464.41		305.21 ± 421.82	
	C/C	143.67 ± 52.71		587.43 ± 471.60		430.36 ± 426.20	

P values correspond to Kruskal-Wallis tests for independent samples. Pc correspond values adjusted for multiple comparisons according the False Discovery Rate (FDR) procedure. K-W test: Kruskall-Wallis test.

**Table 4:** Characteristics of the individuals included in the study.

	Overall patients with allergic rhinitis (n = 515)	Patients with allergic rhinitis plus asthma (n = 366)	Patients with allergic rhinitis alone (n = 149)	Healthy subjects (n = 526)
Women, n (%)	294 (57.1%)	193 (52.7%)	82 (55.0%)	265 (50.4%)
Age ± sd (range)	32.2 ± 15.1 (14–79)	30.1 ± 13.2 (14–79)	32.5 ± 16.5 (14–77)	28.4 ± 12.1 (18–84)
Smokers, n (%)	151 (29.3%)	82 (22.4%)	69 (46.3%)	168 (31.9%)
Familial antecedents, n (%)	206 (40.0%)	155 (42.3%)	51 (34.2%)	0 (0%)
Total IgE (IU/mL) ± sd (range)	254.1 ± 401.5 (0–4800)	287.2 ± 464.1 (0–4800)	205.7 ± 228.4 (4–1469)	not determined
Eosinophilia, n (%)	88 (17.0%)	86 (23.4%)	2 (1.3%)	not determined
Allergic rhinitis type, n (%)	116/239/160 (22.5/46.4/74.4)	62/185/119 (16.9/50.5/32.5)	54/54/41 ^(2)^ (34.2/34.2/27.5)	n.a.
FEV_1_ (% predicted)	n.a.	94 ± 62	n.a.	n.a.
Asthma severity step 1,2,3,4, n (%)	n.a.	106/130/107/23 (29.0/35.5/29.2/6.3)	n.a.	n.a.

Eosinophilia is defined as an eosinophils count over 400/ml or over 6% of white cells. The category smokers includes ex-smokers. Allergic rhinitis type was classified as follows (intermittent, persistent mild and persistent moderate). n.a. Not applicable. FEV: Forced expiratory volume.
