# Supplementary material for: A Nonsynonymous FCER1B SNP is Associated with Risk of Developing Allergic Rhinitis and with IgE Levels
Source: Sci Rep. 2016 Jan 21;6:19724. doi: 10.1038/srep19724 (PMC4726269; doi:10.1038/srep19724)
Supplement: Supplementary Information [file srep19724-s1.doc]

**A NONSYNONYMOUS *FCER1B* SNP IS ASSOCIATED WITH RISK OF DEVELOPING ALLERGIC RHINITIS AND WITH IgE LEVELS**

Gemma Amo, MSc^a^

Jesús García-Menaya, MD, PhD ^b^

Paloma Campo MD, PhD ^c^

Concepción Cordobés, MD ^b^

Mª Carmen Plaza Serón, MsC^c^

Pedro Ayuso, PhD ^d^

Gara Esguevillas, BSc^a^

Miguel Blanca, MD, PhD ^c^

Jose A.G. Agúndez, MD, PhD ^a^

Elena García-Martín, MD, PhD ^a*^

From: ^a^ Department of Pharmacology, Universidad de Extremadura, Cáceres, Spain.

^b^Allergy Service, University Hospital Infanta Cristina, Badajoz, Spain. ^c^Allergy Service, Hospital Carlos Haya, Málaga, Spain. ^d^ Research Laboratory, IBIMA-Málaga University General Hospital, Málaga, Spain.

**^*^Corresponding author**: Prof Elena García-Martín. Dept. Pharmacology. University of Extremadura. Avda de la Universidad s/n, 10071, Cáceres, Spain. E-mail: [elenag@unex.es](mailto:elenag@unex.es)

Supplemental Table S1. Details of the SNPs analyzed in this study.

| Gene | Chromosomal location | dbSNP | Assay ID | MAF (1000 genomes, European individuals) |
| --- | --- | --- | --- | --- |
| *FCER1A* | 1:159253672 | rs2494262 | C____494924_20 | 0.56 |
| *FCER1A* | 1:159258545 | rs2427837 | C__16233438_20 | 0.3 |
| *FCER1A* | 1:159272060 | rs2251746 | Custom-designed | 0.3 |
| *FCER1B* | 11:59856028 | rs1441586 | C___1842226_10 | 0.46 |
| *FCER1B* | 11:59863104 | rs569108 | C____900116_10 | 0.04 |
| *FCER1B* | 11:59863253 | rs512555 | C___7513065_10 | 0.04 |
| *FCER1G* | 1:161184875 | rs11587213 | C__27848237_10 | 0.18 |
| *FCER1G* | 1:161185058 | rs2070901 | C__15867981_20 | 0.27 |
| *FCER1G* | 1:161188936 | rs11421 | C___1841966_1_ | 0.15 |

**dbSNP:** Single Nucleotide Polymorphism Database; **MAF:** Minor allele frequency
